# Unraveling the Link Between Periodontal Disease and High Cholesterol: A Cross-Sectional Study

**DOI:** 10.7759/cureus.43463

**Published:** 2023-08-14

**Authors:** Tooba Zahid Khan, Talha Mobin

**Affiliations:** 1 Dentistry, Combined Military Hospital (CMH) Lahore Medical College and Institute of Dentistry, Lahore, PAK; 2 Dornsife School of Public Health, Drexel University, Philadelphia, USA

**Keywords:** dyslipidemia, national health and nutrition examination survey (nhanes), epidemiology, oral medicine and periodontology, clinical dentistry

## Abstract

Objective: This cross-sectional study aimed to investigate the association between high cholesterol levels and the prevalence of periodontal disease among patients in the United States, utilizing data from the National Health and Nutrition Examination Survey (NHANES) conducted between 2017 and 2020. We hypothesize that patients with high cholesterol levels will have a high prevalence of periodontal disease.

Methods: The study utilized a cross-sectional design, analyzing data from NHANES 2017-March 2020 pre-pandemic survey, a nationally representative survey. The sub-sample consisted of 7,042 participants aged 30 years and older who underwent the NHANES survey questionnaire. Information on high cholesterol levels and periodontal disease was obtained through self-reported data. Statistical analyses, using SAS 9.4 (SAS Institute Inc., Cary, NC), were performed to assess the association between high cholesterol levels and periodontal disease prevalence while controlling the potential confounding variables. Descriptive statistics and multivariable logistic regression were used.

Results: The study included a total of 7,042 participants. The mean age (SD) of the participants was 60.2 (13.7); 54% were females and 46% were males. Out of the total, 23% (1636) of the samples had periodontal disease and 52% (3669) of the participants had high cholesterol levels. Findings indicated a significant association between high cholesterol and periodontal disease (odds ratio, OR = 1.21, 95% confidence interval, CI = 1.06-1.40). Socioeconomic factors such as poverty and education, and other factors such as age, gender, race, smoking, physical activity, BMI, diabetes, hypertension, sugar intake, cholesterol intake, saturated fatty acids intake, and oral hygiene were identified as potential confounders.

Conclusion: This large-scale cross-sectional study investigated the association between high cholesterol and periodontal disease while comprehensively controlling for potential confounding factors. After adjusting for the relevant confounders, we found a weak positive association between high cholesterol and periodontal disease. While these findings provide valuable insights into the interplay between systemic health and oral health, the cross-sectional nature of the study limits the establishment of causality.

## Introduction

In recent years, researchers and healthcare professionals have increasingly recognized that oral health is intricately connected to overall systemic health [1]. Among the various oral health conditions, periodontal disease has emerged as a potential risk factor for numerous systemic diseases, including cardiovascular disease, diabetes, and respiratory ailments [2]. Periodontal disease, a chronic inflammatory condition affecting the supporting structures of the teeth, such as gums and bones, is one of the most prevalent oral health issues globally [3]. Severe periodontitis, which is the sixth most prevalent chronic disease among the general population, affects nearly 750 million people worldwide and is thought to affect people’s chewing ability, nutritional status, and quality of life [4,5]. In the United States alone, it affects approximately 47.2% of adults aged 30 years and older, highlighting its significance as a major public health concern [6].

High cholesterol, on the other hand, has long been recognized as a significant risk factor for the development of atherosclerosis and subsequent cardiovascular events [7]. Atherosclerosis is characterized by the buildup of plaque in the arteries, impeding blood flow and potentially leading to heart attacks, strokes, and other vascular complications [8]. The American Heart Association (AHA) identifies high cholesterol levels as a major modifiable risk factor for cardiovascular disease, underscoring the importance of understanding its potential connections with other health conditions [9].

Over the past few decades, a growing body of evidence has suggested a potential bidirectional relationship between periodontal disease and high cholesterol levels [10,11]. Various studies have explored the mechanisms linking these two seemingly distinct health issues, including shared inflammatory pathways and the role of oral bacteria in systemic circulation [12,13]. However, despite these compelling findings, the exact nature of the relationship remains an area of ongoing research.

To address this knowledge gap, we turn to the National Health and Nutrition Examination Survey (NHANES), a nationally representative survey conducted by the Centers for Disease Control and Prevention (CDC) to assess the health and nutritional status of the U.S. population. The NHANES data offers a unique opportunity to investigate the interplay between periodontal disease and high cholesterol on a large scale, providing valuable insights into potential associations, risk factors, and demographic patterns.

As oral health and cardiovascular health continue to gain recognition as interconnected domains, the results of this study may have far-reaching implications for healthcare providers, policymakers, and individuals alike. By recognizing the links between periodontal disease and high cholesterol, we take a step towards comprehensive healthcare strategies prioritizing oral and systemic wellness, ultimately working towards better overall health outcomes for the population.

To shed light on this relationship, the current study aims to investigate the association between high cholesterol levels and the prevalence of periodontal disease among patients in the United States. We hypothesize that patients with high cholesterol levels will have a high prevalence of periodontal disease. We utilize data from the NHANES conducted between 2017 and 2020, a nationally representative survey that includes comprehensive oral health assessments. By analyzing this rich dataset, we can assess the extent to which high cholesterol levels are linked to periodontal disease prevalence while controlling for relevant confounding factors.

## Materials and methods

Study design and subjects

This is a cross-sectional study using the National Health and Nutrition Examination Survey dataset that was collected during the 2017-March 2020 pre-pandemic period nationwide in the United States. This data is available freely, as open access on the Centers for Disease Control and Prevention's website [14]. The National Health and Nutrition Examination Survey enrolled 0-80 years old subjects. For the sub-study, adults, males and females, 30 years of age and above are used in the sample. The data was freely available on CDC's website and is de-identifiable; hence it was exempt from IRB approval.

Outcome

The outcome variable was periodontal disease. The participants, of all genders, aged 30 years and above in the NHANES survey were explained about periodontal disease and had to self-report it. Specifically, they were told that gum disease is a common problem with the mouth. People with gum disease might have swollen gums, receding gums, sore or infected gums or loose teeth. The question they were asked was “Do you think that you might have gum disease?” People who responded “Yes” to this question were coded as “1” and people who responded “No” were coded as “0”. All missing data and participants who didn’t know if they had gum disease or refused to tell were excluded.

Exposure

The exposure variable was high cholesterol. The participants, of all genders, in the NHANES survey were asked “Have you ever been told by a doctor or other health professional that your blood cholesterol level was high?” Participants who responded “Yes” to this were coded as “1” and participants who responded “No” were coded as “0”. All participants with missing data and participants who didn’t know or refused to tell were excluded.

Confounders

Confounders were identified a priori by consulting the relevant literature. We used diagrams to think about the causal process underlying our hypothesis. We used Daggity software (Tumor Immunology Lab and Institute for Computing and Information Sciences, Radboud University, Nijmegen, Netherlands) to help us identify variables that should not be included as confounders (decedents of the exposure {mediators} or descendants of the outcome or colliders). Through this process, we identified at least one minimal adjustment set required to control for confounding and worked to ensure that a minimal set was in the analyses. To control for confounding in the association between exposure and outcome, the variables age, gender, race/ethnicity, smoking, education, socioeconomic status, physical activity, BMI, diabetes, hypertension, sugar intake, cholesterol intake, saturated fatty acids intake, and oral hygiene were used and adjusted for, which furthermore indicates our minimal adjustment set.

Age is an essential factor to consider as both high cholesterol and gum disease are age-related conditions. Cholesterol levels tend to increase with age due to various physiological changes and lifestyle factors [15]. Similarly, gum disease becomes more prevalent as people age, primarily due to cumulative exposure to risk factors and weakened immune responses [16]. Gender is another critical demographic variable that can influence the relationship between high cholesterol and gum disease. Studies have shown that men and women may have different rates of dyslipidemia and gum disease [17]. Biological and hormonal differences between genders can impact cholesterol metabolism and inflammatory responses in the oral cavity. Different racial and ethnic groups may have varying susceptibilities to high cholesterol and gum disease due to genetic, cultural, and lifestyle factors. For instance, certain ethnicities may have a higher prevalence of dyslipidemia or gum disease due to specific dietary habits [18].

Smoking is a well-established risk factor for gum disease and is known to influence cholesterol levels. Smokers tend to have higher cholesterol levels and an increased risk of gum disease due to the harmful effects of tobacco on oral health [19]. Education and socioeconomic status can impact access to healthcare, dietary choices, and oral hygiene practices. Individuals with higher education and socioeconomic status may be more likely to adopt healthier lifestyles, including better oral hygiene and dietary habits, which could influence both high cholesterol and gum disease risk [20]. Physical activity is associated with improved cardiovascular health and may also influence gum health. Regular physical activity has been shown to positively affect cholesterol levels and reduce the risk of gum disease [21]. BMI is closely related to both high cholesterol and gum disease. Obesity is a risk factor for dyslipidemia and gum disease, and individuals with higher BMI may be more susceptible to both conditions [22].

Diabetes is associated with both high cholesterol and an increased risk of gum disease. Poorly controlled diabetes can lead to dyslipidemia and may also impair the body's ability to combat infections, including those in the oral cavity [23]. Hypertension may be linked to both high cholesterol and gum disease. Studies have shown that individuals with hypertension may have altered lipid profiles and an increased risk of gum disease [24]. High sugar intake is associated with dyslipidemia and can also influence the development of gum disease. Diets high in sugar have been shown to increase cholesterol levels and contribute to gum inflammation and disease progression [25]. The dietary intake of cholesterol could impact both cholesterol levels in the body and the risk of gum disease. Diets high in cholesterol-rich foods may contribute to dyslipidemia and could also influence inflammatory processes in the oral cavity [26]. Saturated fat consumption can impact cholesterol levels and may also affect gum health. A diet high in saturated fats can increase "bad" cholesterol (LDL) levels. Additionally, inflammation caused by high saturated fat intake might influence gum disease risk [27]. Inadequate oral hygiene practices, such as infrequent brushing and flossing, can lead to gum disease. Poor oral hygiene is associated with an increased risk of gum inflammation and periodontitis [28].

Analytic sample and missing data

Among the 24,085 participants who contributed to the data, 7,042 participants were excluded for not having any data related to our research. Further exclusions were made as follows: 256 without an exposure measurement and 263 without an outcome measurement and 9,482 who did not have full covariate data. Following the complete-case analysis approach, the final analytic sample was 7,042 participants (30% of the original sample).

Statistical method

The statistical package used for the analysis was SAS 9.4 (SAS Institute Inc., Cary, NC). Unadjusted descriptive statistics (percentages and means) examined participants' characteristics relative to the exposure (high cholesterol) and outcome (periodontal disease). Multivariable adjusted logistic regression was used to model the relative odds of having periodontal disease. Confounders were selected a priori based on our literature review and directed acyclic graphs. Model 1 was the unadjusted model. Model 2 adjusted for age and gender. Model 3 added race, smoking, diabetes, hypertension, physical activity, total sugar intake, total saturated fatty acid intake, total cholesterol intake, and oral hygiene to age and gender, and Model 4 added BMI, socioeconomic status, and education. A p-value of 0.05 and 95% CI was used to test for statistically significant associations.

## Results

Descriptive results

Demographics and other characteristics of the participants are reported in Table 1. The total number of participants who were included in the analysis was 7,042. Among these 46% were males and 54% were females. The mean age of the participants was 60.2 years. Of the total, 43% of participants were White, 25% were Black, 10% were Mexican, 9% were other Hispanic, 7% were Asian and 6% were multi-race. Of the total, 19% of the participants had a less than high school education, 25% had a high school education, and 56% had a greater than high school education. Of the total, 18% of the participants were poor, 29% were near poor, and 53% were not poor. Of the total, 34% of the participants were diabetic and 63% were hypertensive; 51% of the participants were obese, 32% were overweight, 16% were healthy, and 1% were underweight. Of the total, 34% of the participants did some physical activity, 52% had smoked at least 100 cigarettes in their life, and 36% maintained good oral hygiene compared to 64% who did not. The mean sugar intake of the participants was 102.1 grams. The mean cholesterol intake of participants was 313.5 milligrams and the mean saturated fatty acid intake was 102.1 grams.

**Table 1:** Descriptive characteristics of total participants (N=7,042). N: Number of participants; BMI: Body Mass Index; SD: Standard Deviation; gm: Grams; mg: Milligrams

Characteristics	N	%
Mean Age (SD)	60.2 (13.7)	-
Gender	-	-
Male	3262	46%
Female	3780	54%
Race	-	-
White	3060	43%
Black	1757	25%
Mexican	681	10%
Other Hispanic	664	9%
Asian	493	7%
Multi-Race	387	6%
Education	-	-
Less than high-school	1316	19%
High-school	1802	25%
Greater than high-school	3924	56%
Poverty	-	-
Poor	1292	18%
Near Poor	2055	29%
Not Poor	3695	53%
Diabetes	-	-
Yes	2405	34%
No	4637	66%
Hypertension	-	-
Yes	4436	63%
No	2606	37%
BMI	-	-
Underweight	78	1%
Healthy	1161	16%
Overweight	2242	32%
Obese	3561	51%
Physical Activity	-	-
Yes	2413	34%
No	4629	66%
Smoking (Smoked at least 100 cigarettes in life)	-	-
Yes	3658	52%
No	3384	48%
Oral Hygiene	-	-
Yes	2535	36%
No	4507	64%
Mean Sugar Intake in gm (SD)	102.1 (72.1)	-
Mean Cholesterol intake in mg (SD)	313.5 (250.3)	-
Mean Saturated Fatty Acid intake in gm (SD)	27.1 (16.5)	-

There were 1636 (23%) participants with periodontal disease and 5406 (77%) who did not have periodontal disease. Of the total, 3669 (52%) participants had high cholesterol compared to 3373 (48%) of the participants who did not have high cholesterol levels. These results are presented in Table 2.

**Table 2:** Distribution of participants who had high cholesterol levels and periodontal disease.

	Yes (%)	No (%)	Total
Periodontal Disease	1636 (23%)	5406 (77%)	7042
High Cholesterol	3669 (52%)	3373 (48%)	

Among those who had periodontal disease, 53% had high cholesterol. The mean age of these participants was 58.4; 45% were males and 55% were females. Among the participants with periodontal disease, 43% were White, 25% were Black, 10% were Mexican, 9% were other Hispanic, 7% were Asian and 6% were multi-race. Of the total, 20% of the participants with periodontal disease had a less than high school education, 26% had a high school education, and 54% had a greater than high school education. Among those who had periodontal disease, 22% were poor, 32% were near poor, and 46% were not poor. Among the participants with periodontal disease, 38% had diabetes, 65% had hypertension, 2% were underweight, 17% were healthy, 31% were overweight, and 51% were obese. Among the participants who had periodontal disease, 30% reported doing some physical activity, 56% of the participants also reported smoking at least 100 cigarettes in their life, and 26% of the participants with periodontal disease had good oral hygiene. The mean sugar intake among participants with periodontal disease was 110.4 grams, the mean cholesterol intake was 310.3 milligrams and the mean saturated fatty acid intake was 27 grams. Among those who had high cholesterol, 77% had periodontal disease. The mean age of these participants was 63.8. 49% of them were males and 51% were females. Among the participants with high cholesterol, 46% were White, 25% were Black, 9% were Mexican, 11% were other Hispanic, 6% were Asian and 5% were multi-race. Of the total, 20% of the participants with high cholesterol had a less than high school education, 26% had a high school education and 54% had a greater than high school education. Among those who had high cholesterol, 17% were poor, 31% were near poor and 52% were not poor. Among the participants with high cholesterol, 45% had diabetes and 77% had hypertension. Less than 1% of the participants with high cholesterol were underweight, 13% were healthy, 36% were overweight, and 51% were obese. Among the participants who had high cholesterol, 31% reported doing some physical activity, 55% of the participants also reported smoking at least 100 cigarettes in their life, and 66% of the participants with high cholesterol had good oral hygiene. The mean sugar intake among participants with high cholesterol was 97.6 grams, the mean cholesterol intake was 321.6 milligrams and the mean saturated fatty acid intake was 26.9 grams. These results are given in Table 3.

**Table 3:** Distribution of sample characteristics for total participants (N=7042), stratified by periodontal disease and high cholesterol. n: Number of participants; SD: Standard Deviation; BMI: Body Mass Index; gm: Grams; mg: Milligrams

Characteristics	Periodontal Disease (N, Col%)		High Cholesterol (N, Col%)	
-	Yes (n=1636, 23%)	No (n=5406, 77%)	Yes (n=3669, 52%)	No (n=3373, 48%)
Periodontal Disease	-	-	-	-
Yes	1636 (100%)	5406 (100%)	2808 (77%)	775 (23%)
No	0	0	861 (23%)	2598 (77%)
High Cholesterol	-	-	-	-
Yes	861 (53%)	2598 (48%)	3669 (100%)	0
No	775 (47%)	2808 (52%)	0	3373 (100%)
Mean Age (SD)	58.4 (12.3)	60.8 (14.1)	63.8 (11.9)	56.4 (14.5)
Gender	-	-	-	-
Male	743 (45%)	2519 (47%)	1803 (49%)	1459 (43%)
Female	893 (55%)	2887 (53%)	1866 (51%)	1914 (57%)
Race	-	-	-	-
White	704 (43%)	2356 (44%)	1679 (46%)	1381 (41%)
Black	404 (25%)	1353 (25%)	851 (23%)	906 (27%)
Mexican	159 (10%)	522 (10%)	340 (9%)	341 (10%)
Other Hispanic	151 (9%)	513 (9%)	380 (11%)	284 (8%)
Asian	122 (7%)	371 (7%)	222 (6%)	271 (8%)
Multi-Race	96 (6%)	291 (5%)	197 (5%)	190 (6%)
Education	-	-	-	-
Less than high-school	323 (20%)	993 (18%)	725 (20%)	591 (18%)
High-school	422 (26%)	1380 (26%)	959 (26%)	843 (25%)
Greater than high-school	891 (54%)	3033 (56%)	1985 (54%)	1939 (57%)
Poverty	-	-	-	-
Poor	359 (22%)	933 (17%)	624 (17%)	668 (20%)
Near Poor	521 (32%)	1534 (28%)	1150 (31%)	905 (27%)
Not Poor	756 (46%)	2939 (54%)	1895 (52%)	1800 (53%)
Diabetes	-	-	-	-
Yes	614 (38%)	1791 (33%)	1574 (43%)	
No	1022 (62%)	3615 (67%)	2095 (57%)	
Hypertension	-	-	-	-
Yes	1069 (65%)	3367 (62%)	2841 (77%)	1595 (47%)
No	567 (35%)	2039 (38%)	828 (23%)	1778 (53%)
BMI	-	-	-	-
Underweight	37 (2%)	41 (1%)	11 (<1%)	67 (2%)
Healthy	270 (17%)	891 (16%)	463 (13%)	698 (21%)
Overweight	499 (31%)	1743 (32%)	1308 (36%)	934 (28%)
Obese	830 (51%)	2731 (51%)	1887 (51%)	1674 (49%)
Physical Activity	-	-	-	-
Yes	488 (30%)		1139 (31%)	1274 (38%)
No	1148 (70%)		2530 (69%)	2099 (62%)
Smoking (Smoked at least 100 cigarettes in life)	-	-	-	-
Yes	923 (56%)	2735 (51%)	2014 (55%)	1644 (49%)
No	713 (44%)	2671 (49%)	1655 (45%)	1729 (51%)
Oral Hygiene	-	-	-	-
Yes	433 (26%)	4074 (75%)	2411 (66%)	2096 (62%)
No	1203 (74%)	1332 (25%)	1258 (34%)	1277 (38%)
Mean Sugar Intake in gm (SD)	110.4 (82.3)	99.6 (68.5)	97.6 (66.7)	107 (77.2)
Mean Cholesterol intake in mg (SD)	310.3 (243.1)	314.4 (252.5)	321.6 (255.2)	304.6 (244.7)
Mean Saturated Fatty Acid intake in gm (SD)	27.0 (16.1)	27.1 (16.6)	26.9 (16.0)	27.2 (17)

Primary adjusted analysis

Table 4 displays the progressive adjustment for the covariates in each of the four models. Results of the final model indicate a weak positive association between high cholesterol and periodontal disease. That is, after controlling for all confounders such as age, gender, race, smoking, diabetes, hypertension, physical activity, total sugar intake, total cholesterol intake, total saturated fatty acids intake, oral hygiene, BMI, education, and poverty, the odds of having periodontal disease are 1.21 times the odds in people who have high cholesterol compared to people who do not have high cholesterol level (OR = 1.21, 95% CI = 1.06-1.40). A weaker positive association was found between periodontal disease and high cholesterol level alone (OR = 1.028, 95% CI = 0.920-1.15). After adjusting for age and gender, periodontal disease and high cholesterol had a somewhat stronger association (OR = 1.14, 95% CI = 1.02-1.28) and after adjusting for age gender, race, smoking, diabetes, hypertension, physical activity, total sugar intake, total cholesterol intake, total saturated fatty acids intake and oral hygiene, periodontal disease, and high cholesterol had a somewhat stronger association (OR = 1.18, 95% CI = 1.03-1.35).

**Table 4:** Adjusted OR and 95% CI of the association between periodontal disease and high cholesterol. *Statistically significant result (p-value < 0.05). OR: Odds Ratio; CI: Confidence Interval

Periodontal Disease = Yes				
-		95% CI		
-	OR	Low	High	P-value
Model 1: Unadjusted Model	1.028	0.920	1.15	0.63
Model 2: Adjusted For Age and Gender	1.14	1.02	1.28	0.02*
Model 3: Adjusted For Age, Gender, Race, Smoking, Diabetes, Hypertension, Physical Activity, Total Sugar Intake, Total Cholesterol Intake, Total Saturated Fatty Acids Intake, and Oral Hygiene	1.18	1.03	1.35	0.018*
Model 4: Adjusted For Age, Gender, Race, Smoking, Diabetes, Hypertension, Physical Activity, Total Sugar Intake, Total Cholesterol Intake, Total Saturated Fatty Acids Intake, Oral Hygiene, BMI, Education and Poverty	1.21	1.06	1.40	0.0059*

## Discussion

The observed positive association between high cholesterol and periodontal disease aligns with findings from several previous studies [29,30]. Epidemiological investigations have increasingly explored the relationship between periodontal disease and systemic inflammatory conditions, including dyslipidemia and high cholesterol levels [31].

A comprehensive systematic review conducted by Watanabe et al. provided robust evidence of the association between periodontal disease and elevated cholesterol levels [32]. The review included data from multiple studies, and the pooled results demonstrated that individuals with periodontal disease were more likely to have dyslipidemia and higher cholesterol levels compared to those with healthy periodontal status. The results of this review align with our findings.

Furthermore, a study by Fukui et al. explored the relationship between periodontal disease and lipid profiles in a large sample of middle-aged and elderly individuals [33]. The researchers found that periodontal disease was associated with increased total cholesterol, low-density lipoprotein cholesterol (LDL-C), and triglyceride. These findings suggested that periodontal disease might influence lipid metabolism, contributing to an unfavorable lipid profile associated with a higher risk of cardiovascular diseases.

In a cross-sectional study involving a Korean population, Lee et al. examined the relationship between periodontal disease and lipid profiles [34]. The researchers found that individuals with severe periodontal disease had higher total cholesterol. The association remained significant even after controlling for various lifestyle and metabolic factors, further supporting the link between periodontal disease and dyslipidemia.

In a systematic review and meta-analysis, Chaffee and Weston investigated the impact of periodontitis on lipid profiles and obesity. The meta-analysis included randomized controlled trials that assessed lipid profile changes among periodontal disease patients. The results revealed that periodontitis was associated with high cholesterol levels and obesity [35].

In a cross-sectional study involving patients with generalized severe periodontitis, Andrukhov et al. investigated the serum and saliva levels of nitric oxide (NO) metabolites in periodontal disease and their relationship with serum C-reactive protein (CRP) levels, lipids metabolism, and periodontal disease severity [36]. The researchers found that individuals with severe periodontitis patients exhibited significantly lower serum and saliva levels of NO metabolites and significantly higher LDL, cholesterol, and CRP levels than the control group. The study provided evidence for the interplay between periodontal disease, systemic inflammation, and lipid metabolism.

In an experimental study involving rats, Brito et al. investigated the systemic inflammatory response and cardiovascular changes induced by experimental periodontitis in rats [37]. The researchers induced periodontitis by placing ligatures around the cervix of both sides of the mandibular first molars and maxillary second molars in each male rat. The results indicated that ligature-induced periodontitis reduced endothelium-dependent vasodilatation, a hallmark of endothelial dysfunction. This effect was associated with increased systemic inflammatory markers (interleukin{IL}-6 and CRP), worsening lipid profile, increased vascular superoxide production, and reduced NOS-3 expression. Thus, endothelial dysfunction correlated with the severity of periodontal disease, reinforcing the potential role of inflammation-induced endothelial dysfunction in dyslipidemia development.

Additionally, investigations focusing on inflammatory markers have provided supporting evidence for the link between periodontal disease and dyslipidemia [38]. Elevated levels of pro-inflammatory cytokines, such as IL-6, CRP, and tumor necrosis factor (TNF)-α, have been observed in both periodontal disease and dyslipidemia. These inflammatory mediators play key roles in atherosclerosis development and lipid metabolism regulation, further supporting the potential relationship between the two conditions.

The strengths of this study are that it includes a substantial sample size of 7,042 participants, which enhances the statistical power and generalizability of the results to a broader population. The study analyzed data from the NHANES survey conducted between 2017 and 2020. The extended duration allows for a more robust examination of the association between the prevalence of periodontal disease and high cholesterol, potentially capturing variations over time. The study rigorously controlled for numerous potential confounding variables, such as age, gender, race, smoking, diabetes, hypertension, physical activity, total sugar intake, total cholesterol intake, total saturated fatty acids intake, oral hygiene, BMI, education, and poverty. This comprehensive adjustment increases the validity of the observed association between high cholesterol and periodontal disease. The study population reflects diverse racial, ethnic, educational, and socioeconomic backgrounds, making the findings more applicable and relevant to various populations. The study's results are consistent with previous research that has suggested a potential link between high cholesterol and periodontal disease. This strengthens the credibility of the study's findings.

It is important to acknowledge the limitations of our study. The cross-sectional nature of the NHANES data limits our ability to establish causality or determine the temporal relationship between periodontal disease and high cholesterol levels. Longitudinal studies with a prospective design would provide stronger evidence for understanding the impact of high cholesterol on periodontal disease prevalence. Furthermore, sampling bias was an issue in our study since only 24% of our sample was 30 to 50 years of age. The over-50 age group has a higher prevalence of periodontal disease than the under-50 group. The issue led to an increased number of observations with the outcome but did not affect the relationship between it and the exposure, leading to the conclusion that it was sampling bias, as it was only related to the outcome. Furthermore, residual confounding might have been present. Prescribed cholesterol medication might have confounded the relationship between periodontal disease and high cholesterol levels. Since cholesterol medication has a negative association between cholesterol level and periodontal disease, the measure of association would be biased away from the null. The study relied on self-reported data for variables such as periodontal disease and high cholesterol levels. Self-reporting is subject to recall bias and may be influenced by social desirability bias, potentially impacting the accuracy of the reported information. Objective measures of periodontal disease and high cholesterol levels would enhance the reliability of the findings.

## Conclusions

Periodontal disease and high cholesterol are both widespread health conditions that have attracted significant attention from researchers and healthcare professionals. Our study aimed to investigate the association between high cholesterol and periodontal disease while meticulously controlling for a comprehensive set of potential confounding variables. The results of this large-scale cross-sectional analysis provided valuable insights into the relationship between these prevalent health conditions. After adjusting for all the confounders, we observed a weak positive association between high cholesterol and periodontal disease. The observed weak positive association suggests a potential link between these two health factors; however, it is crucial to acknowledge the complexity of the human body's physiological mechanisms and the influence of various other factors. While this study contributes to our understanding of the interplay between oral health and systemic well-being, additional rigorous research is needed to delineate the precise nature of this association and its underlying mechanisms. As we delve deeper into this intriguing intersection, healthcare professionals can better tailor their preventive and treatment strategies, considering both oral hygiene and cholesterol management to promote holistic patient health.
